# The neuroscience of formal thought disorder - the state of the art

**DOI:** 10.1192/j.eurpsy.2023.341

**Published:** 2023-07-19

**Authors:** E. Dornelles, D. Telles-Correia

**Affiliations:** CLINICA UNIVERSITÁRIA DE PSIQUIATRIA E PSICOLOGIA MÉDICA, FACULDADE MEDICINA UNIVERSIDADE DE LISBOA, Lisboa, Portugal

## Abstract

**Introduction:**

Even though the construct of Formal Thought Disorder (FTD) is an ambiguous and disputed one, it has endured as a fundamental psychopathological concept in the clinical coalface of Psychiatry. FTD can be summarized as a multidimensional concept, which reflects difficulties or idiosyncrasies in thought, language, and communication in general. It is usually subdivided into positive versus negative and objective versus subjective, and it can be a major challenge for both mental health professionals and patients themselves.

**Objectives:**

In this presentation, we aim to explore the latest neuroscientific findings regarding FTD and its putative neurobiological substrate, ranging from the synaptic and neurotransmitter level to the structural and functional one, briefly considering some of the linguistic and neuropsychological implications.

**Methods:**

We conducted a thorough narrative review by researching the Pubmed database using the following search string: “formal thought disorder”[Title/Abstract] and selecting only those articles published after 2010. Afterwards, we summarized the main findings from the gathered information.

**Results:**

Some of the most consistent findings in current meta- and mega-analyses of structural MRI studies in patients with schizophrenia and FTD are volume reductions of regional grey matter in the frontal operculum and the language-related lateral temporal cortices, namely the left superior temporal gyrus and middle temporal gyrus. Another consistent finding is the so-called reversed lateralization of the temporal cortices. Regarding functional MRI studies of FTD, amongst the most common implicated regions are the bilateral superior and middle temporal gyri, the fusiform gyrus and the inferior frontal gyrus. Alterations in the glutamatergic, dopaminergic and serotoninergic neurotransmitter systems have also been linked to FTD.

**Conclusions:**

Many areas of the brain have been implicated in the pathogenesis of FTD, though some more consistently than others. The superior temporal, middle temporal and inferior frontal gyri in particular have repeatedly revealed both structural and functional alterations in patients with FTD. A reversed lateralization has also been observed at both structural and functional levels. The different neurotransmitter systems have also been connected with FTD, with the glutamate system being the one more thoroughly explored. However, the direction of causality between changes in the brain and FTD, and the influence of potential mediators remain largely unknown.

**Disclosure of Interest:**

None Declared

